# Optimized PCR Conditions and Increased shRNA Fold Representation Improve Reproducibility of Pooled shRNA Screens

**DOI:** 10.1371/journal.pone.0042341

**Published:** 2012-08-01

**Authors:** Žaklina Strezoska, Abel Licon, Josh Haimes, Katie Jansen Spayd, Kruti M. Patel, Kevin Sullivan, Katarzyna Jastrzebski, Kaylene J. Simpson, Devin Leake, Anja van Brabant Smith, Annaleen Vermeulen

**Affiliations:** 1 Molecular Biology, Thermo Fisher Scientific, Lafayette, Colorado, United States of America; 2 Victorian Centre for Functional Genomics, Peter MacCallum Cancer Centre, East Melbourne, Victoria, Australia; 3 The Sir Peter MacCallum Department of Oncology, The University of Melbourne, Parkville, Victoria, Australia; National Institute of Health, United States of America

## Abstract

RNAi screening using pooled shRNA libraries is a valuable tool for identifying genetic regulators of biological processes. However, for a successful pooled shRNA screen, it is imperative to thoroughly optimize experimental conditions to obtain reproducible data. Here we performed viability screens with a library of ∼10 000 shRNAs at two different fold representations (100- and 500-fold at transduction) and report the reproducibility of shRNA abundance changes between screening replicates determined by microarray and next generation sequencing analyses. We show that the technical reproducibility between PCR replicates from a pooled screen can be drastically improved by ensuring that PCR amplification steps are kept within the exponential phase and by using an amount of genomic DNA input in the reaction that maintains the average template copies per shRNA used during library transduction. Using these optimized PCR conditions, we then show that higher reproducibility of biological replicates is obtained by both microarray and next generation sequencing when screening with higher average shRNA fold representation. shRNAs that change abundance reproducibly in biological replicates (primary hits) are identified from screens performed with both 100- and 500-fold shRNA representation, however a higher percentage of primary hit overlap between screening replicates is obtained from 500-fold shRNA representation screens. While strong hits with larger changes in relative abundance were generally identified in both screens, hits with smaller changes were identified only in the screens performed with the higher shRNA fold representation at transduction.

## Introduction

In recent years, RNAi screens have become a useful method for identifying genetic regulators of a range of biological processes [1], [2]. A commonly used approach for performing RNAi screens in mammalian cells is arrayed screening, in which individual RNAi triggers (siRNAs, shRNAs, or microRNAs) are distributed across individual wells in multi-well culture plates and phenotypes are screened on a well-by-well basis. An alternative approach is to perform pooled RNAi screens in which hundreds or thousands of different shRNAs are introduced into a population of cells. These cells are then selected for the phenotype of interest and examined for proviral shRNA abundance compared to a control. The main advantage to pooled screening is that the experiments do not require expensive automation, storage of large arrayed RNAi collections or well-by-well analysis. In addition, by using this approach RNAi screening can be applied to study phenotypes that take longer to develop because shRNAs integrate into the genome. Pooled shRNA screens have been successfully used to identify genetic regulators of cell proliferation and survival [3]–[5], tumorigenicity [6]–[8], adhesion [9], migration [10], drug modulation [11]–[13] and even cancer phenotypes in mouse models [14]–[16].

Retroviral and lentiviral shRNA libraries have been used for pooled shRNA screening. With both of these viral delivery methods, cells are transduced and shRNAs stably integrate into the genomes of the host cells. The transduced cells are subsequently subjected to phenotypic selection and/or selective pressure. Cells expressing shRNAs targeting genes involved in the phenotype are enriched or depleted relative to the control population of transduced cells. In order to identify these shRNAs of interest, proviral shRNAs or their associated molecular barcodes are PCR-amplified from genomic DNA (gDNA) isolated from the cell populations, and the relative abundance of the individual shRNAs is compared between control and selected cell populations using custom microarrays [3]–[5], [11], [17], [18] or next generation sequencing (NGS) [19], [20]. Although it is clear that factors influencing each of these experimental steps may have effects on the quality of the screen and the biological significance of the hits obtained from the screen, a thorough analysis of the technical considerations for performing successful and reproducible pooled shRNA screens has only recently begun to emerge [21]–[23].

One of the critical considerations of pooled lentiviral shRNA screening is the extent to which any given shRNA construct in a pooled library will be represented throughout the screening process. It is plausible that identification of shRNAs that are enriched during the selection process would have a less stringent requirement of average fold representation than identification of shRNAs that are depleted during the selection process. The average shRNA fold representation at transduction (the number of independent integrations per shRNA) varies among published screens. For example, while some groups transduce cells with enough viral particles such that each shRNA in the library is represented by at least 1 000 copies [4], [5], [20], [24], other groups have used equivalents of 10 to 20 copies per shRNA [9], [25], [26]. However, a side-by-side comparison of different shRNA fold representations in the context of a biological screen and its implications has not been reported. Furthermore, the requirement for maintaining the shRNA fold representation throughout the experiment including the PCR amplification steps has not been addressed. In particular, the amount of gDNA input in the PCR step (corresponding to the average template copies per shRNA) has not been established.

Here we examined the effects of shRNA fold representation at transduction on the reproducibility of pooled shRNA screening data. We performed viability screens with a library of ∼10 000 shRNAs at two different fold representations (100- and 500-fold at transduction) and report the reproducibility of changes in proviral shRNA abundance between screening replicates determined by microarray and NGS analyses. We show that the technical reproducibility between PCR replicates from a screen can be drastically improved by 1) ensuring that the PCR amplification steps are maintained in the exponential phase and 2) using an amount of gDNA input in the reaction that maintains the average template copies per shRNA that was used during library transduction. Using these optimized PCR conditions, we also show that reproducibility between screening (biological) replicates improves with increased shRNA fold representation at transduction and amplification. This higher reproducibility results in a greater overlap of primary hits between the biological screening replicates when using either analysis method. shRNA hits with smaller fold changes in abundance were identified in screens using higher shRNA fold representation, however shRNA with robust fold changes were generally identified in screens with both low and high shRNA fold representation.

## Results

### PCR amplification affects screen reproducibility

After transduction and integration of shRNA sequence into the cells' genomes, PCR amplification allows for quantitative detection of the relative changes of proviral shRNA sequence or barcode sequence following selection. This integral step of the workflow must maintain shRNA representation and avoid bias. In published shRNA pooled screens amplification conditions are often incompletely reported and the methods of amplification vary widely. Therefore, a preliminary study was designed to assess the effects of amplification on pooled RNAi screening reproducibility, thus leading to optimization of PCR conditions for minimal introduction of bias. For this evaluation, we used the Decode library of annotated genes which contains 10 353 shRNAs targeting 6 792 human genes and created both a reference sample and a test sample. The reference sample was generated by amplifying molecular barcodes from the plasmids used to create the Decode library. The test sample was generated by amplifying molecular barcodes from HeLa cells infected with the Decode lentiviral particles. HeLa cells were transduced with the Decode library at 100-fold average shRNA representation and were selected with puromycin before gDNA was isolated. Comparison of the reference and test samples from selected cells simulates a viability screen scenario where the abundance of some shRNAs has decreased in the test sample due to effects on cell viability. The correlation between replicates of each of the samples (reference and test) as well as their log ratios can be examined. PCR amplification was performed on both reference and test samples with input gDNA corresponding to 50 or 150 template copies per shRNA for each sample, and all samples were amplified for 25 or 30 cycles. To ensure the PCR reaction conditions were similar between the experiments, when higher gDNA input was required, additional PCR reactions were performed (4 and 12 reactions with ∼800 ng of gDNA per reaction for 50 and 150 template copies, respectively). This experiment resulted in eight unique samples, each of which was prepared in technical duplicate. Reference samples, T_0_, were labeled with Cy3 and test samples, T_1_, were labeled with Cy5. Microarray analysis by competitive hybridization was performed comparing T_1_/T_0_ for each replicate to evaluate the PCR reproducibility as a function of cycle number and amount of gDNA input.

**Figure 1:**
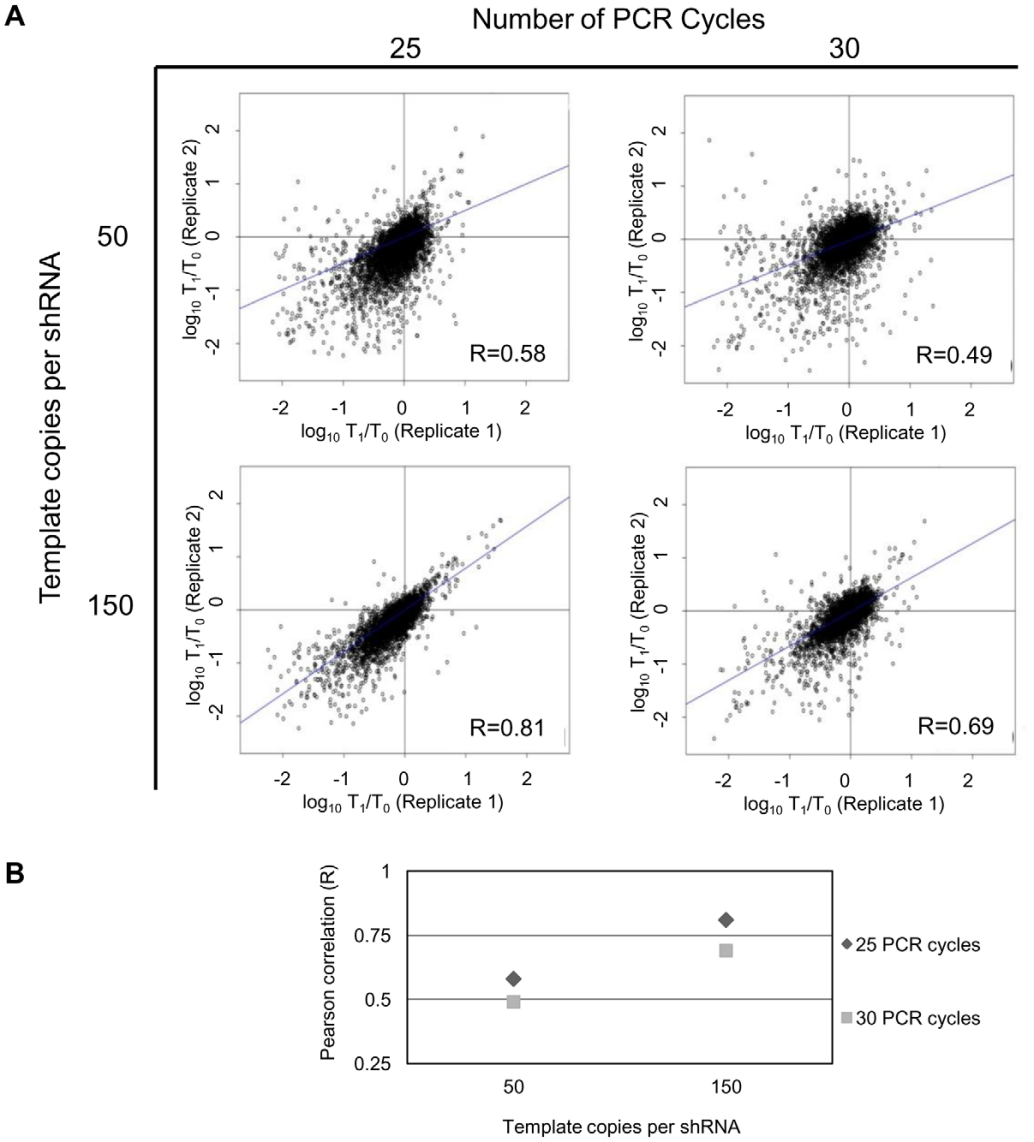
PCR template amount and cycle number affect screen technical data reproducibility. A. Scatter plots of log_10_(T_1_/T_0_) of technical PCR replicates. T_1_ sample was generated from gDNA isolated from HeLa cells transduced with a pooled library of 10 000 lentiviral shRNAs with an average shRNA representation of 100-fold and cultured under puromycin selection. T_0_ reference sample was generated from a pool of plasmids used to create the lentiviral library. Barcode sequences were PCR amplified in technical duplicates for 25 and 30 cycles from the gDNA or the plasmid pool with input DNA corresponding to 50 and 150 template copies per shRNA. Analysis of shRNA abundance in T_1_ samples compared to T_0_ samples was performed using competitive microarray hybridization. Pearson correlation values for each graph are indicated in the corner of each scatter plot. B. Graphical representation of the Pearson correlation values as a function of template copies per shRNA and number of PCR cycles.

The reproducibility between T_0_ and T_1_ replicates were examined and showed excellent correlation when 150 template copies per shRNA were used and good (but lower) correlation when 50 template copies were used (Table S1). PCR cycle number had a small impact on reproducibility of the T_0_ and T_1_ replicates. However, because pooled screening data relies on detecting relative changes in abundance, the Pearson correlation R values for the log_10_(T_1_/T_0_) of replicates were examined for the different samples to evaluate experimental noise and reproducibility. Examination of the R values for the log_10_(T_1_/T_0_) of replicates reveals that the correlation of PCR replicates was improved by decreasing the amplification cycle number from 30 to 25 cycles and increasing template input from 50 to 150 template copies per shRNA (Figure 1, Table S1). Indeed, a substantial improvement in reproducibility was observed when both PCR conditions tested (cycle number and template input) were considered, resulting in an increase in the Pearson correlation from 0.49 (at 30 cycles and DNA input corresponding to 50 template copies per shRNA) to 0.81 (at 25 cycles and DNA input corresponding to 150 template copies per shRNA). This data demonstrates that data reproducibility in screens can be improved substantially by optimization of PCR amplification.

The libraries used in pooled screening exhibit various ranges in shRNA abundance. For the reference, the shRNA abundance is expected to be a reflection of plasmid library composition. For the test sample, a larger range of abundance is expected since it depends on both library composition and biological effects from shRNAs in transduced cells. Ideally, the protocol should not introduce bias (for example by increasing the range of shRNA abundance); otherwise the changes detected in shRNA abundance might not be biologically relevant. Therefore, we examined the effects of PCR amplification on the range of shRNA abundance for reference plasmid (T_0_) and the transduced test sample (T_1_) by looking at the minimum fold difference between the least and most represented shRNAs for 70% of the shRNA population (Figure S1). Similar to the Pearson correlation values, we find that the abundance range increases for both samples with increased PCR cycle number and decreased template copies per shRNA. The fold change in shRNA abundance is particularly pronounced for the transduced test sample (T_1_), where the difference between the least and most represented shRNAs changes from 14- to 69-fold. For this reason the impact of PCR amplification on pooled shRNA screening data reproducibility needed to be systematically examined further. First, the number of amplification cycles during PCR was examined because amplification is most quantitative during the exponential phase of the reaction where the copy number is doubled at each cycle for 100% efficient reactions [27]. Second, the amount of gDNA input (number of template copies per shRNA) used for PCR was examined because the number of template copies should be representative of the average copy number of the shRNA population upon transduction.

### Identification of the exponential phase of the PCR amplification

The exponential phase of the polymerase chain reaction is the most uniform and quantitative phase of the reaction, which is necessary for accurate and reproducible analysis of shRNA abundance in the pooled shRNA screening workflow. At the same time, the amplification reaction must yield sufficient product for downstream analysis of relative shRNA abundance using platforms such as microarray and NGS. To satisfy both requirements of reproducible and sufficient amplification, it is necessary to identify the transition from exponential to linear PCR amplification.

**Figure 2:**
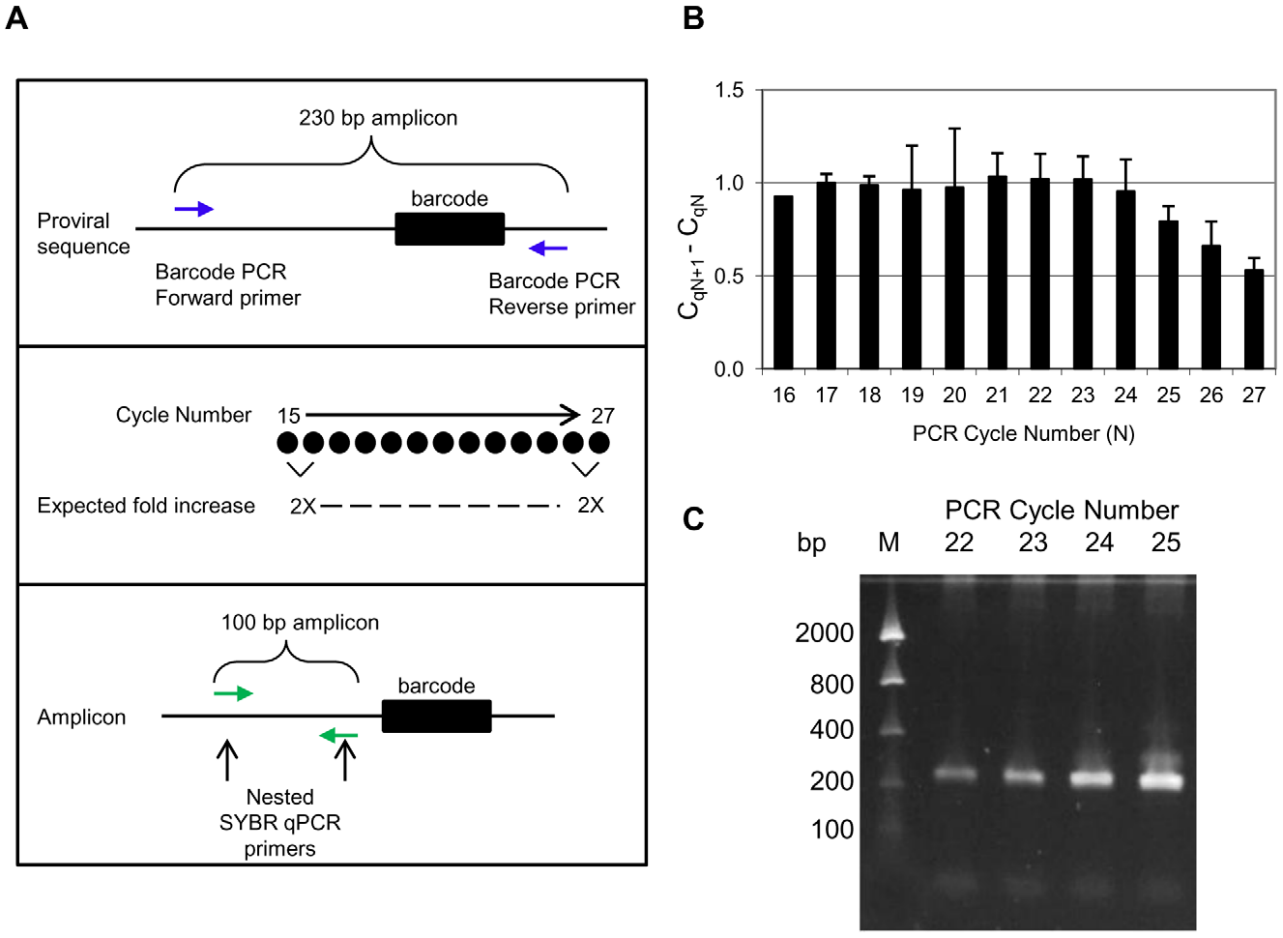
Identification of the exponential phase during PCR amplification of barcode sequences. A. Schematic of the strategy used to identify the transition point from exponential to linear PCR amplification. gDNA isolated from HEK293T cells transduced with the pooled shRNA library were amplified in replicate PCR reactions. A replicate reaction was stopped at each cycle from 15 to 27 cycles. Subsequently, PCR products were used as templates for SYBR qPCR reactions using nested primers targeting a common sequence (outside of the barcode region) to examine the ΔC_q_ between cycles. B. Difference of C_q_ obtained in the qPCR on diluted amplicons from every cycle of the Phusion HS II polymerase PCR reaction (C_qN+1_−C_qN_) as a function of the Phusion PCR cycle number (N). C. Gel analysis of the PCR product generated from amplification cycles 22 to 25. Sizes of DNA bands in DNA marker (lane M) are indicated on the left.

To identify this transition point, gDNA was isolated from HEK293T cells transduced with the Decode library and replicate PCR reactions were performed using 825 ng gDNA per reaction, a high-fidelity polymerase and primers flanking the barcode sequence. The replicate reactions were stopped at different cycle numbers spanning a range from 15–27 cycles. In order to assay the cycle number at which accumulation of the PCR product indicates a transition from exponential to linear amplification, and ultimately the plateau phase of the reaction, the PCR products were diluted and used as templates for SYBR qPCR reactions using nested primers targeting a common region (Fig. 2A). Assuming 100% efficiency of the high-fidelity amplification reaction, a doubling of products is expected from one cycle to the next. If amplicons from such adjacent cycle reactions are diluted equally and used as templates for a 100% efficient SYBR reaction, these templates are expected to produce SYBR qPCR C_q_ values with a difference of one. The PCR cycle where the difference between SYBR qPCR C_q_ of that cycle and the following cycle is less than 1 represents the point at which the high-fidelity amplification is no longer doubling product from one cycle to the next (no longer the exponential phase).

The data indicate that the high-fidelity amplification remains in the exponential phase until cycle 23, after which there is a gradual reduction of the SYBR ΔC_q_ values between subsequent amplification cycles (Fig. 2B). The data also show that the amplification reaction is near 100% efficient during the exponential phase since the ΔC_q_ between cycles up until cycle 23 is one. In addition, the yield of the PCR product generated after 23 cycles of amplification is sufficient for purification, labeling and microarray hybridization and it can be clearly detected when analyzed by gel electrophoresis (Fig. 2C).

### Examination of the effect of shRNA fold representation at transduction on screen reproducibility

**Figure 3:**
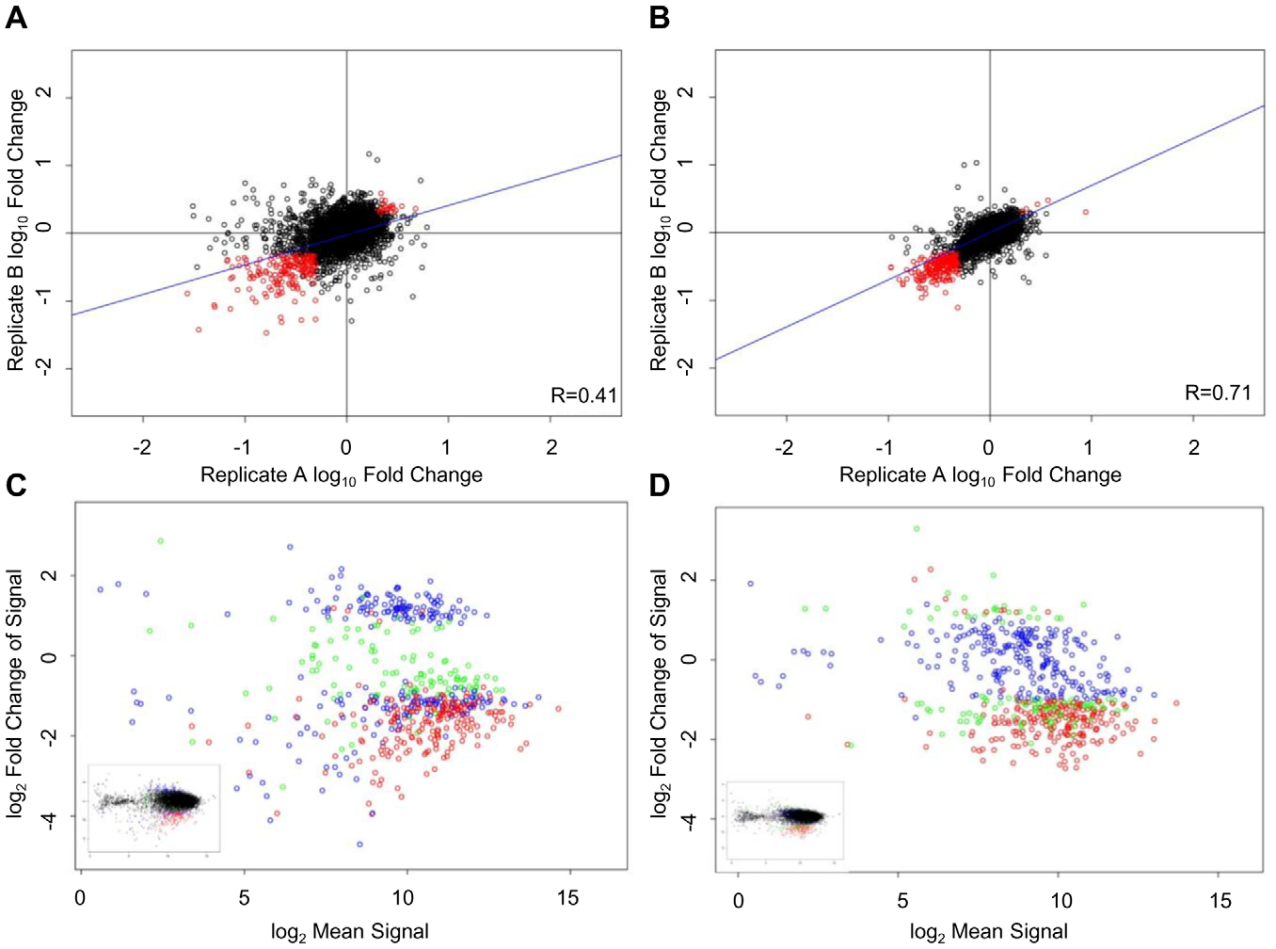
Effect of shRNA fold representation on reproducibility of a HEK293T viability screen using microarray analysis. Viability screens in HEK293T cells were performed using an average shRNA fold representation of either 100 (S100) or 500 (S500) at transduction and the change of the relative abundance of shRNA in T_1_ compared to T_0_ was analyzed by competitive microarray hybridizations. Scatter plot of log_10_(T_1_/T_0_) of the biological replicates of the S100 (A) and S500 (B) screens are shown with Pearson correlation values indicated in the corner of each plot. Probes were filtered to remove those which did not pass T_0_ signal>two-fold median background. Primary hits (probes that passed fold change criteria of (T_1_/T_0_) greater than two and FDR rate of ≤0.05 in both screening (biological) replicates are depicted in red. Signal (log_2_Mean Signal) for S100 (C) and S500 (D) screens are plotted as a function of log ratio (log_2_(T_1_/T_0_)). Primary hits are color coded with hits identified in both S100 and S500 screens (red), hits identified in the S100 screen only (blue) and hits identified in the S500 screen only (green). The complete data set is presented in the small insert.

The optimal cycle number for PCR amplification of the barcode, determined above, was used in a viability screen using HEK293T cells in which shRNA fold representation during transduction was varied to examine its effect on screen reproducibility. A screen was chosen that uses experimental conditions similar to those applied in a viability study performed by Schlabach and co-workers [4]. Cells were transduced with the Decode library at either 100- or 500-fold shRNA representation (termed S100 and S500 screens, respectively) in biological duplicates (A and B) at a low MOI, such that most of the cells should have a single viral integration. A reference sample (T_0_) was obtained from transduced cells after four days of puromycin selection, and a test sample (T_1_) was harvested following 14 additional days of puromycin selection (Figure S2). Amplification of the barcode sequence was performed in technical replicates on gDNA isolated from each biological screen replicate. The amount of gDNA input used for PCR amplification corresponded to 100 template copies per shRNA for the S100 screen and 500 template copies per shRNA for the S500 screen, thus maintaining the fold shRNA representation during transduction in the amplification step. The volume of the PCR reaction and the concentration of the PCR reaction components were kept constant and the number of PCR reactions was increased to account for amount of input gDNA. Microarray analysis was performed to identify shRNAs that were enriched or depleted in T_1_ compared to T_0_ samples. In the S100 and S500 screens, respectively, 96.2% and 97.3% of the shRNAs were identified as present in the reference samples (T_0_). Log_10_(T_1_/T_0_) data reproducibility and ranges of shRNA abundance were examined as a function of shRNA fold representation at transduction for both technical (PCR) replicates and biological replicates (Figure 3A and 3B, Figure S3).

The technical replicate data produced using the optimized PCR conditions show very good correlation with similar R values (ranging from 0.65 to 0.73) for both screens (S100 and S500) indicating that technical reproducibility is not affected by the shRNA fold representation at transduction (Figure S4). Furthermore, the data show that the optimal amplification conditions result in robust technical reproducibility in a screening example. However, the effect of shRNA fold representation during transduction on biological reproducibility has a profound impact on reproducibility. As the fold shRNA representation at transduction increased, the correlation between biological replicates improved considerably (from R = 0.41 for the S100 screen, Figure 3A, to R = 0.71 for the S500 screen, Figure 3B). In contrast, increasing gDNA input for the S100 screen to 250 template copies per shRNA only gave a modest improvement of the biological data correlation (from 0.41 to 0.46, data not shown). Reducing the gDNA input for the S500 screen from 500 to 250 template copies per shRNA reduced the biological data reproducibility (from R = 0.71 to R = 0.64, data not shown). These changes, although small, underscore the importance of maintaining the template copies per shRNA in PCR to represent the coverage during transduction. Using optimal amplification conditions, the range of shRNA abundance is substantially improved to less than 10 minimum fold difference between the least and most represented shRNAs for the 70% of the shRNA population for the reference (T_0_) and test (T_1_) HEK293 samples (Figure S3). In summary, this analysis demonstrates that increasing the shRNA fold representation at transduction and maintaining the shRNA fold representation during amplification substantially improves the reproducibility of the screening data.

While correlation between biological replicates is an important measure of screening data quality, identification of hits (potential gene targets) is the primary goal of a screen. Therefore, the data was analyzed to identify primary hits. These are the shRNAs that affected cell viability either negatively or positively (decreased or increased shRNA abundance, respectively) in the selected cell population (primary hits and data shown in Table S2). We then examined whether the primary shRNA hits were reproducibly identified in screens which used different shRNA fold representation at transduction. shRNA hits (shown in red, Figure 3A and B) were selected using a fold change criteria (T_1_/T_0_) of greater than two and false discovery rate (FDR) of ≤0.05. The S100 screen identified 633 and 634 hits in replicates A and B, respectively with an overlap of 263 hits (42%) between replicates (Table 1). The higher shRNA representation S500 screen identified 337 and 352 hits in replicates A and B, respectively, with an overlap of 237 hits (70%) between replicates (Table 1). While approximately the same number of overlapping hits between biological replicates were identified for both the S100 and S500 screens, the S500 screen which had a higher shRNA fold representation at transduction (and maintained that fold representation at PCR amplification) resulted in a higher percentage of reproducible hits between biological replicates. Examination of the hits identified by the microarray analysis in the S100 and S500 screens (by combining the biological and technical replicates of each screen in the hit analysis) showed an overlap of 66% percent of hits (Table 1), suggesting that even though the S100 screen was less reproducible than the S500 screen, a large proportion of the hits identified at 500-fold were also identified in the screen with 100-fold shRNA representation.

**Table 1:** Hit reproducibility between experiments.

Experiment 1	Number of hits	Experiment 2	Number of hits	Number of overlapping hits	Percent Overlap
Biological replicates at a given shRNA fold representation					
**S100 A - Microarray** 1	633	**S100 B - Microarray** 1	634	**226**	36%
**S500 A - Microarray** 1	337	**S500 B - Microarray** 1	352	**234**	69%
Comparison between different fold representation					
**S100 (A & B) - Microarray** 2	450	**S500 (A & B) - Microarray** 2	320	**209**	65%
Biological replicates at a given shRNA fold representation					
**S100 A - NGS** 3	170	**S100 B - NGS** 3	112	**73**	65%
**S500 A - NGS** 3	237	**S500 B - NGS** 3	232	**170**	73%
Comparison between different fold representation					
**S100 (A & B) - NGS** 4	319	**S500 (A & B) - NGS** 4	524	**260**	82%
Comparison between readout methods					
**S100 (A & B) - Microarray** 2	450	**S100 (A & B) - NGS** 5	244	**161**	66%
**S500 (A & B) - Microarray** 2	320	**S500 (A & B) - NGS** 5	394	**250**	78%

1 Microarray experiment where two technical replicates were combined for each biological replicate in Rosetta Resolver to identify hits (Fold change >2, p≤0.05).

2 Microarray experiment where two technical and two biological replicates (A & B) were combined for either S100 screen with 100 copies per shRNA in PCR or S500 screen with 500 copies per shRNA in PCR using Rosetta Resolver to identify hits (Fold change >2, p≤0.05).

3 Next generation experiment where one biological replicate (no technical replicates) was analyzed using DESeq to identify hits (Fold change >2, p≤0.05).

4 Next generation experiment where two biological replicates (A & B) were combined for either S100 screen with 100 copies per shRNA or S500 screen with 500 copies per shRNA using DESeq to identify hits analyzed using DESeq to identify hits (Fold change >2, p≤0.05).

5 Next generation experiment where two biological replicates (A & B) were combined for either S100 screen with 100 copies per shRNA or S500 screen with 500 copies per shRNA using DESeq to identify hits analyzed using DESeq to identify hits (Fold change >2, p≤0.05). For comparison, hits were filtered to only include hits that were detectable on the microarray.

Visualization of the hits by log intensity ratio (M) as a function of average log intensity (A) or MA plot illustrates the association of the phenotypic strength and hit identification in the screens (Figure 3C and 3D). While hits with robust fold change are generally identified in both screens, the hits that result in a lower fold change could be lost when lower shRNA fold representation is used at transduction. For example, the hits identified only in the S500 screen but not in the S100 screen (green points in Figure 3D) generally show log ratio values close to the cut-off line (modest change in abundance). The same hits, although not identified in the S100 screen, have log ratio values bordering the selected two-fold cut-off (Figure 3C). Further, the hits identified only in the S100 screen are hits with both increased and decreased abundance (blue points in the MA plot in Figure 3C) and in the majority of cases, these hits did not change in abundance in the S500 screen (Figure 3D). A possible explanation for this could be that a higher percent of primary hits in the S100 screen are false positives.

As NGS is also a commonly used readout for pooled shRNA screening analysis, the above described representation experiment was also analyzed using this approach to determine if the same trends could be observed as with microarray analysis. The PCR amplification for NGS was performed on the same S100 and S500 gDNA samples from the above described HEK293T viability screen, using primers that amplify the shRNA region of the integrated sequences. The transition from exponential to linear amplification was also determined for PCR with these primers (Figure S5). The mean percent recovery (shRNAs with 50 or more alignments) was 90% (standard deviation of 1.3%) for the S100 and S500 samples.

**Figure 4:**
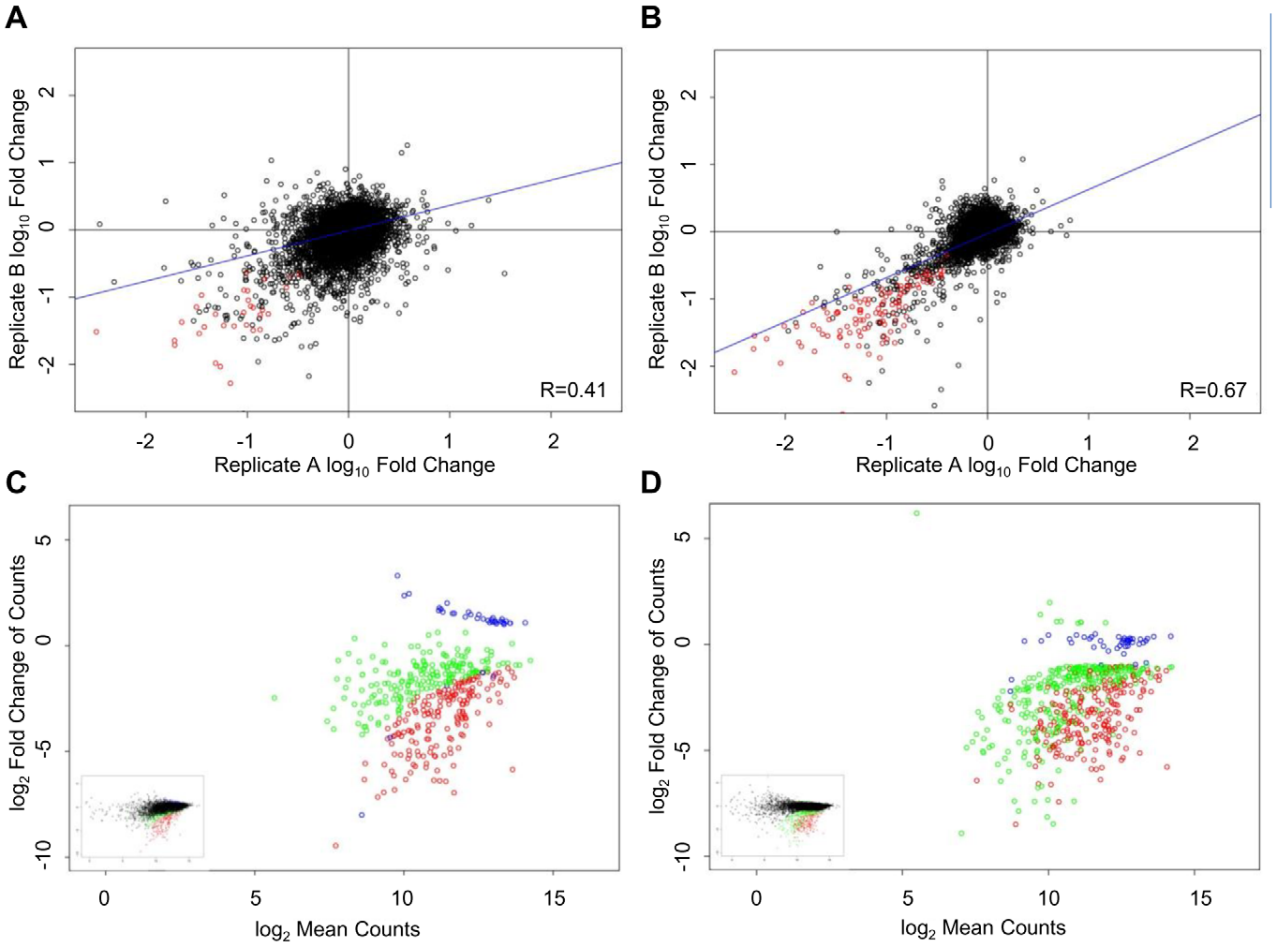
Effect of fold representation of shRNA at transduction on HEK293T viability screen reproducibility using NGS analysis. Viability screens performed in Figure 3 analyzed by NGS. Scatter plot of log_10_(T_1_/T_0_) of the biological replicates of the S100 (A) and S500 (B) screens are shown with Pearson correlation values indicated in the corner of each plot. Primary hits (shRNA that passed fold change criteria of (T_1_/T_0_) greater than two and FDR rate of ≤0.05 in both screening (biological) replicates) are depicted in red. Signal (log_2_Mean Counts) for S100 (C) and S500 (D) screens are plotted as a function of log ratio (log _2_(T_1_/T_0_)). Primary hits are color coded with hits identified in both S100 and S500 screens (red), hits identified in the S100 screen only (blue) and hits identified in the S500 screen only (green). The complete data set is presented in the small insert.

Log_10_(T_1_/T_0_) data reproducibility between the biological replicates was examined as a function of shRNA fold representation at transduction (Figure 4A). Similar to the microarray data, the NGS data showed better reproducibility with higher shRNA fold representation at transduction. The Pearson correlation coefficient of the S500 screen for replicates A and B was 0.67 compared to 0.41 for the S100 screen (Figure 4A and 4B). Examination of the range of shRNA abundance shows less than 10 minimum fold difference between the least and most represented shRNAs for the 70% of the shRNA population for the reference (T_0_) and test (T_1_) screen HEK293 samples, except the S100 T_1_ samples showing a 12-fold difference (Figure S3).

shRNA hits from the NGS analysis were selected using a fold change criteria of (T_1_/T_0_) greater than two and FDR of ≤0.05 (shown in red, Figure 4A and 4B). For the S100 screen, 170 and 112 hits were identified in replicates A and B, respectively, with an overlap of 73 hits (65.2%) between replicates (Table 1). With the higher shRNA representation S500 screen, 237 and 232 hits were identified in replicates A and B, respectively, with an overlap of 170 hits (73.3%) between replicates (Table 1). While the percentage of hit overlap between biological replicates for S100 was high, this percentage further increased for the S500 biological replicates (73.3% compared to 65.2%). In addition, the higher shRNA fold representation at transduction resulted in a higher number of reproducible primary hits between biological replicates (170 compared to 73 hits).

Examination of the hits identified by NGS analysis in both the S100 and S500 screens (by combining the biological replicates of each screen in the hit analysis) showed a relatively high overlap of 83% (Table 1). Similar to the microarray data, visualization of the overlap of NGS hits identified in the S100 and S500 screens in MA plots demonstrated even more clearly that the hits with lower fold change are the ones lost when screening at the lower shRNA fold representation (Figure 4C and 4D). For example, the hits identified only in the S500 screen but not in the S100 screen (green points in Figure 4D) show log ratio values close to the cut-off line in the majority of cases. The same hits, although not identified as hits in the S100 screen, preferentially show log ratio values that are bordering the selected cut-off criteria (Figure 4C). In contrast, there are fewer hits identified only in the S100 screen (blue points in Figure 4C), and these particular shRNAs did not change in abundance in the S500 screen (Figure 4D).

In summary, both microarray and NGS analysis of the pooled shRNA cell viability screen show that higher biological data reproducibility is obtained when screening with higher average shRNA fold representation. However shRNAs that change abundance reproducibly in biological replicates (primary hits) could be identified when screening with both 100- and 500-fold shRNA representation, with a higher percentage of overlap between screening replicates when screening at the higher fold shRNA representation.

## Discussion

Pooled shRNA screening data quality is greatly affected by the screening process as well as by shRNA abundance analysis methods during the deconvolution step. We demonstrate here that PCR amplification can be a substantial source of screening data variability and that optimization of the amplification step dramatically improves the reproducibility of shRNA abundance changes during the pooled shRNA screening process. We were able to significantly improve the reproducibility between technical PCR replicates from a pooled shRNA screen by 1) keeping the PCR amplification in the exponential phase and 2) increasing the reaction volume (by using multiple reaction tubes) to accommodate the larger gDNA input necessary to maintain the representation of shRNAs integrated in the genome.

Hoshiyama and co-workers [23] recently reported variability resulting from the PCR amplification step of pooled shRNA screening and recommended a sample preparation method that involves multiple steps including enrichment of the integrated shRNA sequences from the gDNA and several enzymatic steps during the preparation of the half-hairpin sample for NGS analysis. These multiple steps resulted in increased technical reproducibility [23]. In contrast, our method relies on a simple PCR amplification protocol performed in multiple reaction tubes. Our protocol allows for improvement in reproducibility while minimizing the number of manipulations that can introduce additional bias. A similar PCR amplification strategy for maintaining the shRNA representation has recently been described [22]. In our approach, the PCR steps were also optimized for exponential amplification and the Illumina-adapted primers used for our NGS sample preparation were more than 100 nucleotides away from the stem of the hairpin. Similar to previous reports [28] we found that primer location substantially improved the uniformity of PCR amplification, compared to primers that amplify the half-hairpin (data not shown).

Having optimized PCR amplification conditions for shRNA abundance deconvolution analysis we further examined the effects of the average shRNA fold representation during pooled screening on data reproducibility. Our data shows that increasing shRNA fold representation at transduction from 100- to 500-fold and maintaining the average template copies per shRNA during PCR amplification substantially improves the reproducibility of pooled screening data. A Pearson correlation of the ratio of the shRNA abundance between the screen and reference sample for biological replicates demonstrated this improved reproducibility. The improved reproducibility was also observed in the primary hits identified between biological replicates. Notably, increasing the shRNA fold representation during screening helped to identify hits with smaller fold changes. The reproducibility and size of the hit lists generated from our screens can be used as a proxy for comparing the specificity (1-false positive rate) and sensitivity (1-false negative rate) between pooled screens. A higher percentage of overlapping hits (S500 compared to S100, Table 1) infers that the specificity has increased since overlapping hits are less likely to be due to chance. A larger hit list that maintains a similar percentage of overlapping hits (S500 compared to S100 NGS, Table 1) infers that the sensitivity has increased since there are more reproducible hits with the same percentage of non-overlapping hits. While other reports describe pooled screening readout experiments on samples with simulated decreased shRNA copy number [22], [28] to determine sensitivity, here abundance of integrated shRNAs are examined in a biological screen. This scenario provides a more realistic estimate of biological variance which is essential in determining significantly enriched or depleted shRNAs. Follow up validation with individual shRNAs from the list of hits identified and confirmation of gene involvement in the phenotype could be used to estimate absolute sensitivity and specificity of the screen but was beyond the scope of this study. However, some of these hits have previously been confirmed in published pooled shRNA screens [4] (Table S3).

The number of published pooled shRNA screens using NGS as a readout method is rapidly outpacing that of microarrays. In our study we observed that in general, biological reproducibility improved upon increasing shRNA fold representation, and this was observed through both microarray and NGS analysis. Of note, there is a higher dynamic range in the log ratio data in the NGS analysis (compare Figures 3 and 4). Additionally, the NGS data potentially produced fewer false positive hits in the screen with lower shRNA fold representation compared to the microarray data (compare Figure 3C and 4C). In the NGS experiment, only 23% of the hits from the S100 screen do not appear in the S500 screen (59 of the 260 hits in S100 (A & B) are not present in S500 (A & B)). In the microarray experiment, 54% of the hits from the S100 screen do not appear in the S500 screen (241 out of 450 hits in S100 (A & B) are not present in S500 (A & B)). Since the S500 screen was found to be more biologically reproducible than the S100 screen, one can infer that the hits from the S100 (A & B) analysis that are missing in the S500 (A & B) analysis are likely false positives (Table 1).

Variations between microarray and NGS hit lists may be explained by differences in the sensitivity, dynamic range and technical reproducibility of the two technologies and the use of distinct computational models for determining hits. We analyzed the NGS data and microarray data using different software suites. The NGS data was analyzed using DESeq [29] which models the discrete shRNA counts using a negative binomial distribution. The microarray data, on the other hand, was analyzed using Rosetta Resolver which models the continuous signal of shRNA levels using a normal distribution. The differences in the techniques used by the software to estimate the mean and variance of these models, as well as the statistical tests used to determine significantly enriched or depleted shRNAs may also contribute to the variation in the hit lists. Despite these differences in analysis software, NGS has been shown to have higher sensitivity, higher dynamic range and better technical reproducibility than microarray [30]. These performance differences likely also contribute to the more reproducible hit list obtained with NGS. In addition to these performance benefits, NGS also has the distinct advantage over microarray analysis of being able to sequence any library without having to produce a custom array. The cost of NGS experiments is also declining rapidly and with the added flexibility of multiplexing, it is possible to have many samples run on the same lane, thus even further reducing costs.

Given that our data demonstrates that the reproducibility of pooled screening data increases with the increase of shRNA fold representation at transduction, a reasonable recommendation would be to perform screens at high fold representation. However, the requirement for increasing shRNA fold representation and template copies in the PCR step in order to maintain that high shRNA fold representation has profound logistical consequences for experimental design. Specifically, if we compare the requirements for generating a single replicate of the S100 and S500 experiments where the pool size was approximately 10 000 shRNA, the S100 transduction required 4×10^6^ cells in one 10 mm plate while the S500 transduction required 2×10^7^ cells in five 10 mm plates. Similarly, in the S100 experiment where 6.6 µg gDNA was required for amplification, eight separate PCR reactions were run, while the S500 experiment required 40 PCR reactions. Considering that two or three biological replicates of any screen is required at minimum, scaling the experiment to have a higher shRNA fold representation may become even more challenging, especially for cells that are more difficult to transduce (and for which you will likely need substantially more viral particles) or when the cells of interest are difficult to obtain or culture in large numbers. Additionally, the shRNA fold representation requirements are guided by the type of screen itself. For example, in negative selection screens where the goal is to identify shRNAs that cause cells to become depleted relative to the population as a whole, an ample representation of each shRNA helps to ensure that there is a sufficient window for detection of changes in shRNAs representation after selection. In positive selection screens, on the other hand, where the goal is to identify individual shRNAs that provide a particular advantage to cells under a given selective pressure, identification of enriched shRNAs would not have such strict requirements on shRNA fold representation.

Several strategies can be used to obtain biologically meaningful data from pooled shRNAs screens. Our data demonstrate that even at lower shRNA fold representation, if the PCR reaction is optimized, the percent of primary hits that can reproducibly be identified among biological replicates is high (Table 1). In addition, the use of smaller shRNA pools (for example, 1 000 shRNAs per pool), can also make attaining sufficient fold coverage more feasible while still designing a reasonably sized experiment that will produce biologically relevant data [13], [31], [32].

While RNAi screening using pooled shRNA reagents is a powerful tool for studying biological pathways in mammalian cells, it is important that these screens have a high level of reproducibility in order to identify meaningful primary hits. Here we show that we have successfully optimized protocols for pooled shRNA screening using both microarray and NGS analysis methods. Using these optimized protocols for PCR amplification and increased shRNA fold representation, highly complex shRNA pools can be used successfully to reproducibly identify changes in shRNA abundance during screening. By designing screens that incorporate an understanding of the technical parameters that affect the reproducibility of data from a pooled shRNA screen, researchers will have more confidence in the biological significance of primary hits from a screen, thereby enabling the identification of novel gene targets or pathways that play a role in their phenotype of interest.

## Materials and Methods

### Cell culture

The human embryonic kidney cell line HEK293T (Thermo Scientific, Huntsville, AL) and the human cervical carcinoma cell line HeLa (American Type Culture Collection, Manassas, VA) were propagated and maintained in growth media containing: DMEM High Glucose, with sodium pyruvate supplemented with 10% FBS, 100 U/ml penicillin, 100 µg/ml streptomycin and 200 mM L-glutamine (Thermo Scientific, Logan, Utah), unless otherwise stated.

### Pooled lentiviral shRNA transductions for examination of PCR amplification effects on screen reproducibility

For the T_1_ sample, transductions were performed using pool VIA001 from the lentiviral-based Decode Annotated Genes Library (Thermo Scientific, Huntsville, AL) in HeLa cells. The VIA001 pool is comprised of 10 317 lentiviral shRNA constructs targeting 6 587 human genes. Transductions were performed in 150 mm plates such that each shRNA was represented with an average of 100 copies using an MOI = 0.3 for median single copy integration of each shRNA. Pooled viral particles diluted in 9 ml of transduction media (DMEM with no serum or antibiotics) were added to HeLa cells seeded on the previous day at a density of 5.5×10^6^ cells, followed by incubation for 6 hours at 37°C. Subsequently, 20 ml of growth media was added to the cells followed by incubation at 37°C for an additional 72 hours, at which point cells were selected by propagating in growth media supplemented with 2 µg/ml of puromycin for 14 days. Cells were passaged 1∶4 when 95% confluency was reached. For the T_0_ reference sample, plasmid DNA was used from the VIA001 pool.

### Pooled lentiviral shRNA viability screen

The lentiviral shRNA screen was performed using pool VIA002 from the Decode Annotated Genes Library (Thermo Scientific, Huntsville, AL). The VIA002 pool is comprised of 10 353 lentiviral shRNA constructs targeting 6 792 human genes. Transductions for the screen performed at 100 copies per shRNA were performed in 100 mm plates such that each shRNA was represented with an average of 100 independent integrations per shRNA using an MOI = 0.3 for median single copy integration of each shRNA (S100 screen). For the screen performed at 500 copies per shRNA, cells were transduced at 100 copies per shRNA in five separate 100 mm plates for a total of 500 independent integrations per shRNA and then cells were combined prior to collection at the indicated time points (S500 screen). We performed transductions for both screens in biological duplicate following manufacturer's recommendations. Pooled viral particles diluted in 3 ml of transduction media (DMEM media with no serum or antibiotics) were added to HEK293T cells seeded on the previous day at a density of 2.0×10^6^ cells per 100 mm plate, followed by incubation for 4 hours at 37°C. Growth media (10 mL) was then added to the cells followed by incubation at 37°C for an additional 48 hours, at which point growth media was supplemented with 5 µg/ml of puromycin for selection. After 96 hours of growth under selection, cells were lifted using 1 ml of Trypsin (0.25%, Thermo Scientific, Logan, Utah) and were then divided into reference (T_0_) and test (T_1_) conditions. For the test group, 3.5×10^6^ transduced cells were re-plated on 150 mm plates and propagated under puromycin selection for 14 days. Cells were passaged as needed and maintained at a minimum of 3.5×10^6^ per plate. Remaining cells which were not re-plated were collected for the T_0_ time point. Nuclei were isolated from both T_0_ and T_1_ samples following manufacturer's protocols for the DNeasy Blood and Tissue kit (Qiagen, Hilden, Germany) and then frozen until ready for gDNA isolation.

### gDNA isolation

gDNA was isolated from transduced cells using the DNeasy Blood and Tissue kit (Qiagen, Hilden, Germany) following the manufacturer's protocol. gDNA was isolated from 5×10^6^ HeLa cells, and different amounts of gDNA were, used in the PCR to maintain shRNA representation at 50 and 150 copies per shRNA. gDNA was isolated from 20×10^6^ and 100×10^6^ HEK293T cells for S100 and S500 experiments to accommodate the different experimental replicates. Purified gDNA was evaluated for quality and yield by spectrophotometry using a Nanodrop 1000 (Thermo Scientific, Wilmington, Delaware).

To calculate the amount of gDNA that corresponds to a specific representation of each shRNA present for the screen, we first determined the mass of the human genome by using the number of base pairs (bp) present in the human genome, the average mass of a single base pair and Avogadro's constant:


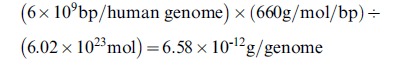


Depending on how many copies of each shRNA were to be represented in the purified gDNA, we used the result above and the total number of desired shRNA integrations assuming a single integration per genome (i.e. approximately 10 000 shRNA per pool at 100 copies each is equivalent to 1×10^6^ shRNA integrations) to calculate the amount of gDNA PCR input.


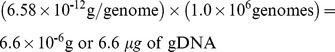


### PCR amplification of the barcode region

Amplification of the barcode region for microarray analysis of transduced HeLa cells was performed using manufacturer protocols and primers provided with Decode Annotated Genes Library for negative selection. 3.3 µg and 9.9 µg of gDNA or 13 pg and 39 pg of plasmid DNA (corresponding to an average representation of 50 template copies per shRNA and 150 template copies per shRNA, respectively) was amplified in technical replicates using 96-well plates such that the total gDNA input was distributed across multiple wells with each well containing 825 ng of gDNA or 3.25 pg of plasmid DNA. Each 50 µl reaction contained 200 µM dNTP, 0.3 µM negative selection primers, 0.5 M betaine and 1 µl KOD HotStart Polymerase (0.02 U/µl final concentration). gDNA was amplified using the following PCR conditions: 94°C for 3 minutes followed by 25 or 30 cycles of 94°C for 35 seconds, 62°C for 35 seconds and 72°C for 1 minute. Individual reactions were then combined according to samples and replicates and the presence of a 250 base pair amplicon was confirmed by agarose gel electrophoresis. Samples were purified using the Wizard SV Gel and PCR Clean-Up System (Promega, Madison, WI) following manufacturer protocols for PCR purification. The purified material was evaluated by a Nanodrop 1000 spectrophotometer for yield and quality.

For the cell viability screen in HEK293T cells, amplification of the barcode region for microarray analysis was performed using Phusion® Hot Start II High-Fidelity DNA Polymerase (Thermo Scientific, Vantaa, Finland) and Decode negative selection primers. Representation of shRNA at transduction was maintained for PCR by amplifying a total of 6.6 µg gDNA for the 100 shRNA screen (S100) or 33 µg for the 500 shRNA screen (S500). Amplification was performed for both biological and PCR technical replicates using 96-well plates, as described in the previous experiment. Reactions were carried out in 50 µl volumes each containing the following reaction components: 10 µl 5× Phusion® HF Buffer, 1 µl 10 mM dNTPs (200 µM final), 5 µl betaine (0.5 M final), 5 µl (each) negative selection primers (0.5 µM final for each primer), 2 µl Phusion Hot Start II Polymerase (0.08 U/µl final), 825 ng gDNA, and PCR grade water. Thermal cycler PCR conditions were 98°C for 3 minutes followed by 23 cycles of 98°C for 10 seconds, 65°C for 15 seconds and 72°C for 15 seconds. Individual reactions were combined and purified, as described previously.

### Identification of the exponential phase of the PCR reaction

PCR was performed as described previously with Phusion Hot Start II polymerase using gDNA isolated from the HEK293T 100 shRNA screen. An optimal concentration of gDNA template in the PCR reaction is 825 ng per 50 µl reaction and further increasing the gDNA was found to inhibit to the amplification (data not shown). Multiple reactions are needed to accommodate larger amounts of gDNA. For example, if one shRNA is integrated per genome, 825 ng gDNA corresponds to 125 000 template total shRNA copies per reaction (one reaction would correspond to 12.5 template copies per shRNA for a library of 10 000 shRNA). Eight reactions (6.6 µg) would be required to maintain 100 template copies per shRNA for a library of 10 000 shRNA.

Twelve replicate reactions containing 825 ng gDNA were amplified and each carried out to a different cycle number from 15–27. Each replicate reaction vessel was placed on ice immediately after the designated number of cycles completed to arrest the reaction. 10 µl of product from each reaction was analyzed using agarose gel electrophoresis. An aliquot of each product was serially diluted 25 000-, 100 000- and 400 000-fold in water. An aliquot from each dilution of each PCR replicate served as template for SYBR qPCR reactions that were prepared using Absolute Blue qPCR SYBR Green master mix (Thermo Scientific, Epsom, UK) and primers that amplify common sequence of the shRNA barcode PCR products (For- 5′caaggggctactttaggagcaa, Rev- 5′aatttataccattttaattcagctttg), generating a product of 127 bp. Quadruplicate 10 µl reactions were prepared by stamping reactions from a 96-well plate into a 384-well plate four times using a Matrix PlateMate 2×3 liquid handler (Thermo Scientific, Hudson, New Hampshire). Each 10 µl 384-well reaction consisted of the following: 5 µl Absolute Blue qPCR SYBR Green master mix (Thermo Scientific, Epsom, UK), 1.33 µl template, 70 nM each forward and reverse primers and nuclease-free water. Thermal cycling was performed and fluorescence data was collected using a LightCycler® 480 (Roche, Indianapolis, IN). Thermal cycling parameters were as follows: 95°C for 15 minutes followed by 40 cycles of 95°C for 15 seconds, 60°C for 30 seconds, 72°C for 30 seconds. Following amplification, a dissociation curve was performed, collecting data from 60°C to 95°C. C_q_ values were determined using default settings of the LightCycler® 480 Software Abs Quant/Fit Points method.

C_q_ values of quadruplicates were averaged and grouped into three data sets according to template dilution factor. The absolute difference of the average C_q_ values between subsequent Phusion PCR cycles within each data set was obtained and averaged across the three different dilution data sets. These values were then corrected for the efficiency of the SYBR qPCR primers in the following manner: first, an efficiency correction factor, E_c_, was calculated using E_c_ = log_(1+(%Efficiency/100))_ 2. For example, for 65% efficient primers E_c_ = log_1.65_ 2 = 1.38. Then, the mean C_q_ difference across the three data sets was divided by the efficiency correction factor to obtain the corrected mean C_q_ difference (C_qN+1_−C_qN_). These values were plotted against Phusion PCR cycle number.

### Microarray hybridization

Purified PCR products containing the barcode region were labeled with either cyanine-3 (Cy3) or cyanine-5 (Cy5) dye using the Genomic DNA Enzymatic Labeling Kit (Agilent Technologies, Santa Clara, CA) and competitively hybridized to Decode barcode microarrays using manufacturer protocols provided with the Decode Annotated Genes Library for negative selection. For each sample, 500 ng of purified PCR product was used for T_0_ (Cy3) or T_1_ (Cy5) sample labeling with subsequent purification. Purified Cy3-labeled T_0_ and Cy5-labeled T_1_ samples were combined and prepared for hybridization using the CGH & ChIP-on-ChIP Hybridization kit (Agilent). Samples were hybridized to microarrays for 17 hours at 65°C and 20 RPM. Microarrays were washed with Agilent CGH/ChIP-on-ChIP wash buffers and immediately scanned at 635 nm and 532 nm using 5 µm resolution with an Agilent Microarray Scanner (Agilent Technologies, Santa Clara, CA).

### Sample preparation for NGS

gDNA isolated from biological replicates from both screens (100 or 500 copies per shRNA) was amplified using custom designed PCR primers with sequences to anneal to the Illumina flow cell (Oligonucleotide sequences © 2006–2008 Illumina, Inc. All rights reserved, Forward- 5′- aatgatacggcgaccaccgagatctacaccggtgcctgagtttgtttgaa, Reverse- 5′- caagcagaagacggcatacgagatggcattaaagcagcgtatccac) that generated a ∼600 base pair amplicon containing the hairpin structure. The forward and reverse PCR primers used for hairpin amplification were designed to contain vector specific sequence along with sequence obtained from Illumina's paired-end primers, the latter of which enabled analysis by the HiSeq 2000 sequencer (Illumina, San Diego, CA). Reactions were carried out in 50 µl volumes each containing the following reaction components: 10 µl 5× Phusion® HF Buffer, 1 µl 10 mM dNTPs (200 µM final), 5 µl betaine (0.5 M final), 5 µl (each) Illumina adapted negative selection primers (0.5 µM final for each primer), 2 µl Phusion Hot Start II Polymerase (0.08 U/µl final), 825 ng gDNA, and PCR grade water. Amplification was performed with the following thermal cycling conditions: 98°C for 3 minutes followed by 24 cycles of 98°C for 10 seconds, 64°C for 15 seconds and 72°C for 15 seconds. PCR product purification was performed using the Promega kit (as described above) followed by elution of the purified PCR samples in EB buffer (Qiagen, Hilden, Germany). Purified samples were submitted for NGS analysis to the Colorado Initiative in Molecular Biotechnology Next-Generation Genomics Facility. Each sample was run on a separate lane using a custom primer 5′- gaaggctcgagaaggtatattgctgttgacagtgagcg (annealing immediately adjacent to the hairpin sequence) for single end reads and produced an average of 89 million 50 base pair reads per lane. Although the number of reads equates to 8 900 reads per shRNA, 1 000 reads per shRNA is more than sufficient for a differential expression experiment.

### Data Analysis

Microarray images were imported into Feature Extraction 9.5.1 (Agilent Technologies, Santa Clara, CA) for quantification of processed signal values following background subtraction. Extraction of data for each probe was performed using extraction protocols and grid files provided with Decode microarrays. Microarray data was imported into Rosetta Resolver (v7.2.2.0). For differential expression analysis, fold changes and p-values were computed for each two-channel experiment using Rosetta's standard ratio experiment pipeline. Rosetta performs a two-sided, two-sample t-test on the means of the probe intensities corresponding to a single clone in each channel. The null hypothesis is that there is no fold change between the clones in the two channels. Only probes that were expected to be in the pool (7 228 of 10 353) were exported and Benjamini-Hochburg Multiple Test Correction [33] was applied to the p-values. Hits were classified as any clone that had an absolute fold change of two or greater and had a multiple-test corrected p-value of 0.05 or lower. For characterizing the reproducibility of replicates the Pearson correlation was computed on the log ratios of each replicate in R (v2.13.11).

To determine presence of an shRNA in the microarray experiments, a one-sample t-test was performed on the background subtracted intensities of the technical replicates for each experiment. The alternative hypothesis is that the true mean is greater than 0. Any shRNA with a p-value of less than 0.05 was considered present.

The minimum range of abundance was computed by selecting 70% of the shRNA population such that the fold difference between the least and most abundant shRNA clone in the set is minimized. This metric describes the distribution of shRNA abundance in each sample.

NGS reads were aligned using Bowtie (v0.12.7) [34]. A Bowtie reference was created using the 10 353 clones expected to be in the pool. When comparing the NGS data with the microarray analysis, only the 7 228 clones which had probes in the microarray were used. The –v 0 option was used to ensure that only perfect matches to the reference clones were tallied. The differential expression analysis was performed using DESeq (v1.4.1) which is an R (v2.13.11) package, part of the Bioconductor (v2.10.0) framework [35]. DESeq uses a model based on the negative binomial distribution to estimate the significance of the fold change. DESeq also applies Benjamini-Hochberg Multiple test correction to the computed p-values. As with microarray data, hits are classified as any clone that had an absolute fold change of two or greater and a multiple-test corrected p-value of 0.05 or lower. For characterizing the reproducibility of replicates the Pearson correlation was computed on the log ratios of each replicate in R (v2.13.11). To determine presence of an shRNA in NGS experiments, any shRNA with more than 50 alignments was considered present.

## Supporting Information

Figure S1 **Effects of PCR amplification on the range of shRNA abundance.** As a measure of the range of shRNA abundance in the population, the minimum fold difference of shRNA abundance between the least and most represented shRNAs for 70% of the shRNA population was examined for the reference plasmid (T_0_) and the transduced test sample (T_1_). It is shown as a function of template copies per shRNA in the PCR amplification and number of PCR cycles. Two replicate PCR amplifications are shown in black and white bars.
(TIF)

Figure S2 **Scheme of the viability screen in HEK293T cells.** Schematic of the viability screens performed using an average fold shRNA representation of either 100 (S100) or 500 (S500) at transduction. Cells were cultured with puromycin-supplemented media for four days to select for populations of cells with integrated viral sequences; a portion of these cells were harvested from each screen for the reference samples (T_0_) and remaining cells were cultured for an additional 14 days under selection before harvesting the test samples (T_1_). gDNA was isolated from T_0_ and T_1_ samples and barcodes were PCR amplified and labeled for competitive hybridization microarray analyses or shRNA were PCR amplified with Illumina adapted primers and analyzed by NGS.
(TIF)

Figure S3 **The range of shRNA abundance in the HEK293T screen samples.** As a measure of range of shRNA abundance in the population, the minimum fold difference of shRNA abundance between the least and most represented shRNAs for 70% of the shRNA population was examined for the reference (T_0_) and test (T_1_) screen HEK293 samples. It is shown as a function of shRNA fold representation at transduction (S100 or S500 screens) and the type of analysis, microarray (MA) or next generation sequencing (NGS).
(TIF)

Figure S4 **Scatter plots of log_10_(T_1_/T_0_) microarray data from the technical (PCR) replicates of the S100 and S500 viability screens.** Amplification of the barcode sequence was performed in technical replicates (1 and 2) on gDNA isolated from each biological screen replicate (A and B). The amplification was limited to the exponential phase of PCR and the amount of input gDNA used corresponded to 100 template copies per shRNA for the S100 screen and 500 template copies per shRNA for the S500 screen. Pearson correlation values (R) for each graph are indicated in the boxes. Probes were filtered to remove those which did not pass a signal cutoff of greater than 2-fold median background in the T_0_ samples.
(TIF)

Figure S5 **Identification of the exponential phase of PCR amplification of the shRNA sequences for NGS sample preparation.** A. Schematic of the strategy used to identify the transition point from exponential to linear PCR amplification with the Illumina adapted primers that amplify the full-hairpin region. Replicate PCR reactions were prepared to amplify gDNA isolated from HEK293T cells transduced with the pooled shRNA library. A replicate reaction was stopped at the stated cycles. Subsequently, PCR products were used as templates for SYBR qPCR reactions using nested primers targeting a common sequence to examine the ΔC_q_ between every other cycle. B. Graphical representation of the ΔC_q_ for the qPCR reactions performed using diluted PCR samples (ΔC_qN+2_−ΔC_qN_) as a function of the Phusion PCR cycle number (N).
(TIF)

Table S1 **PCR conditions affect reproducibility**
(DOCX)

Table S2 **List of primary hits identified in the HEK293 viability screen.**
(XLSX)

Table S3 **List of primary hits identified in the HEK293 viability screen and overlap with the literature.**
(XLSX)
