# Supplementary figures and images for: Optimized PCR Conditions and Increased shRNA Fold Representation Improve Reproducibility of Pooled shRNA Screens
Source: PLoS One. 2012 Aug 1;7(8):e42341. doi: 10.1371/journal.pone.0042341 (PMC3411659; doi:10.1371/journal.pone.0042341)

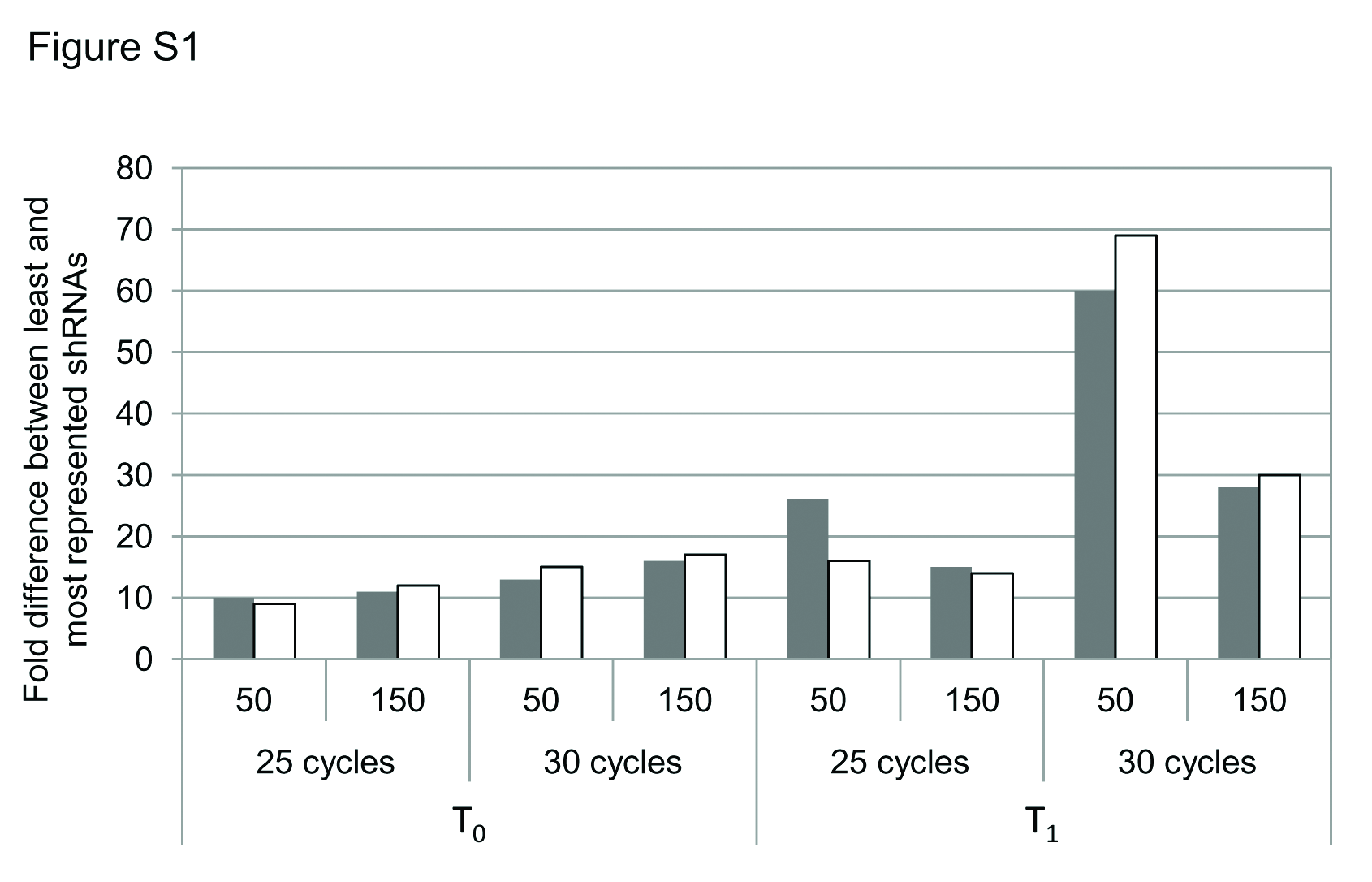

Supplement: Figure S1 — Effects of PCR amplification on the range of shRNA abundance. As a measure of the range of shRNA abundance in the population, the minimum fold difference of shRNA abundance between the least and most represented shRNAs for 70% of the shRNA population was examined for the reference plasmid (T0) and the transduced test sample (T1). It is shown as a function of template copies per shRNA in the PCR amplification and number of PCR cycles. Two replicate PCR amplifications are shown in black and white bars. (TIF) [file pone.0042341.s001.tif]

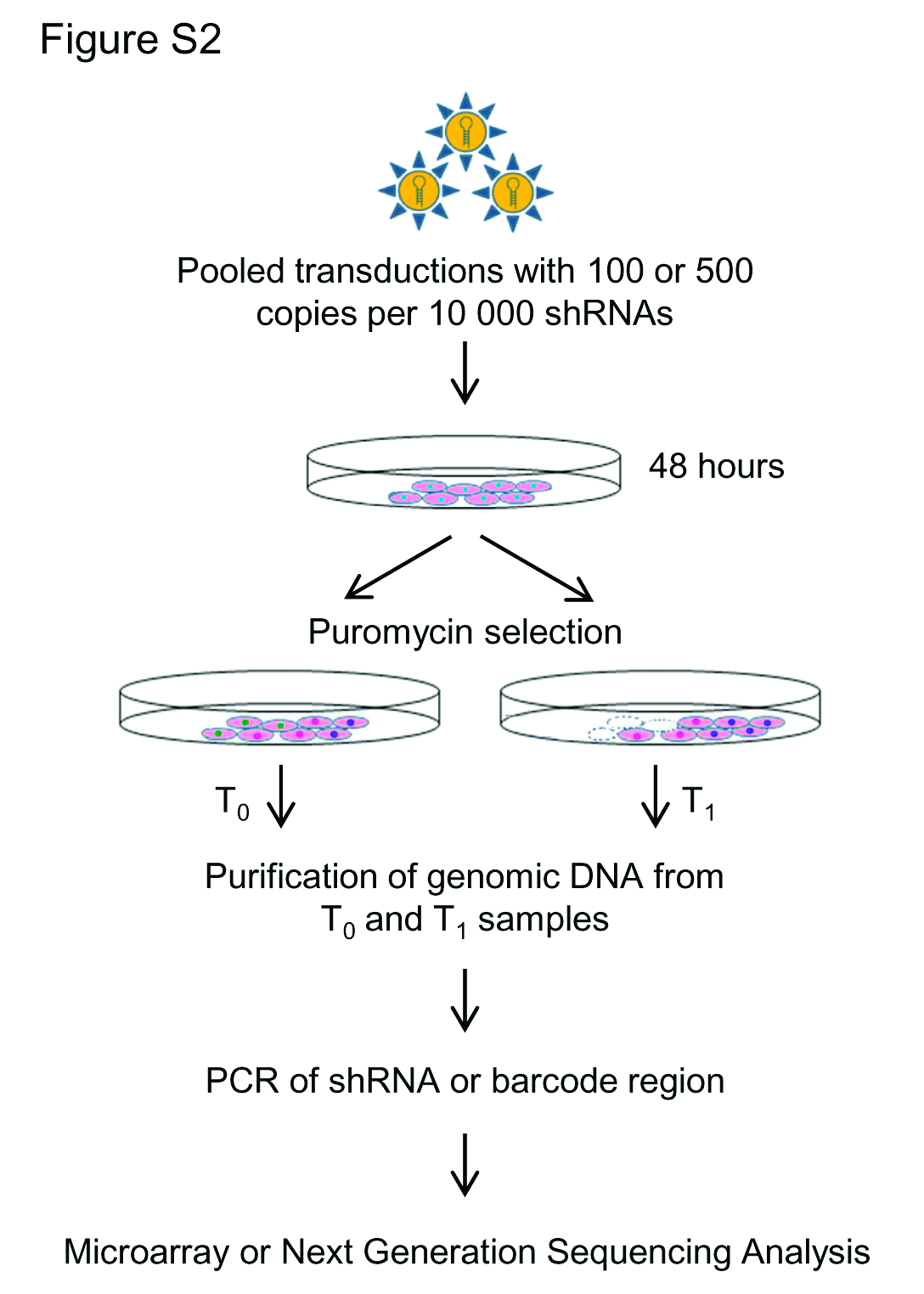

Supplement: Figure S2 — Scheme of the viability screen in HEK293T cells. Schematic of the viability screens performed using an average fold shRNA representation of either 100 (S100) or 500 (S500) at transduction. Cells were cultured with puromycin-supplemented media for four days to select for populations of cells with integrated viral sequences; a portion of these cells were harvested from each screen for the reference samples (T0) and remaining cells were cultured for an additional 14 days under selection before harvesting the test samples (T1). gDNA was isolated from T0 and T1 samples and barcodes were PCR amplified and labeled for competitive hybridization microarray analyses or shRNA were PCR amplified with Illumina adapted primers and analyzed by NGS. (TIF) [file pone.0042341.s002.tif]

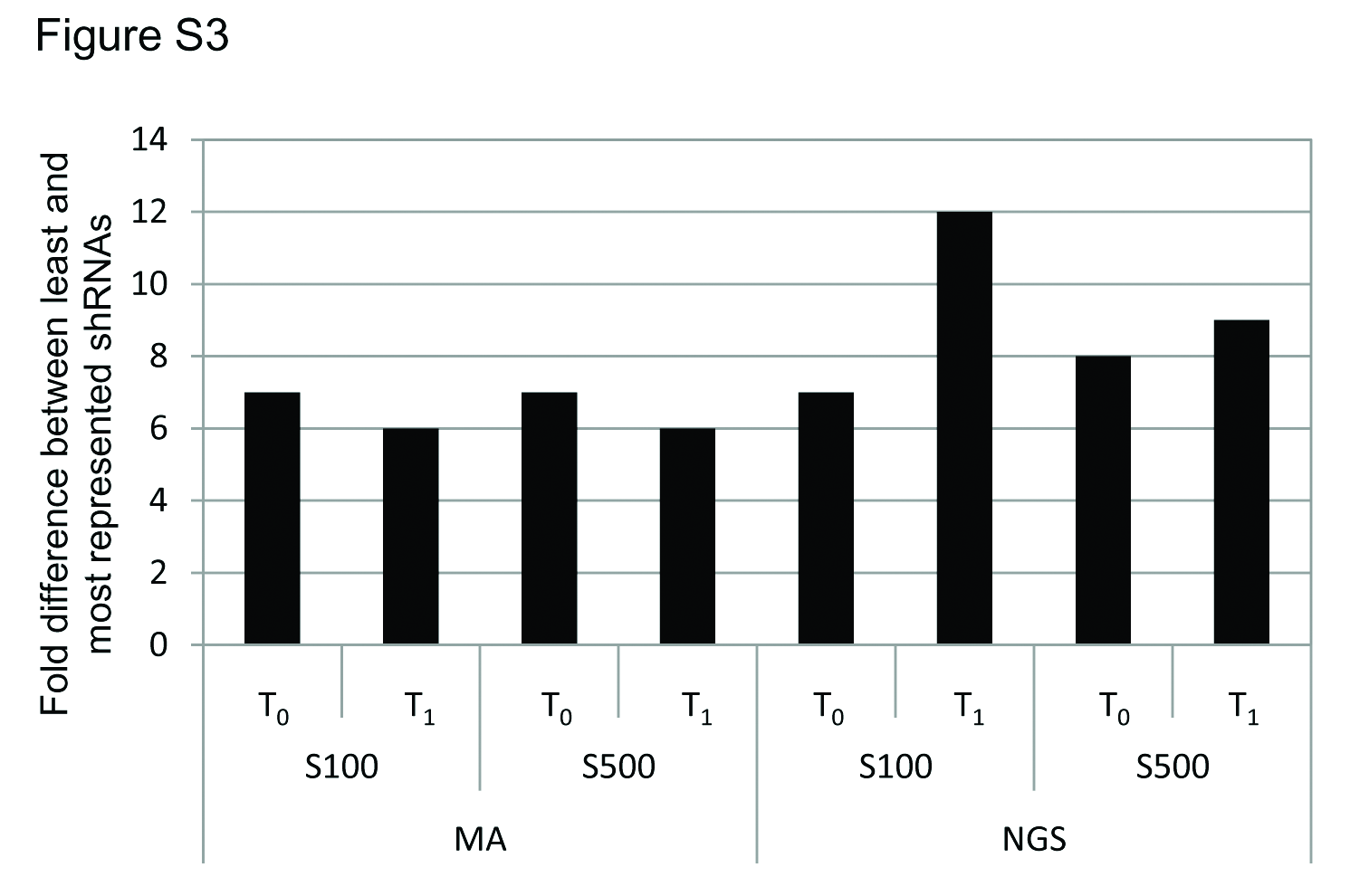

Supplement: Figure S3 — The range of shRNA abundance in the HEK293T screen samples. As a measure of range of shRNA abundance in the population, the minimum fold difference of shRNA abundance between the least and most represented shRNAs for 70% of the shRNA population was examined for the reference (T0) and test (T1) screen HEK293 samples. It is shown as a function of shRNA fold representation at transduction (S100 or S500 screens) and the type of analysis, microarray (MA) or next generation sequencing (NGS). (TIF) [file pone.0042341.s003.tif]

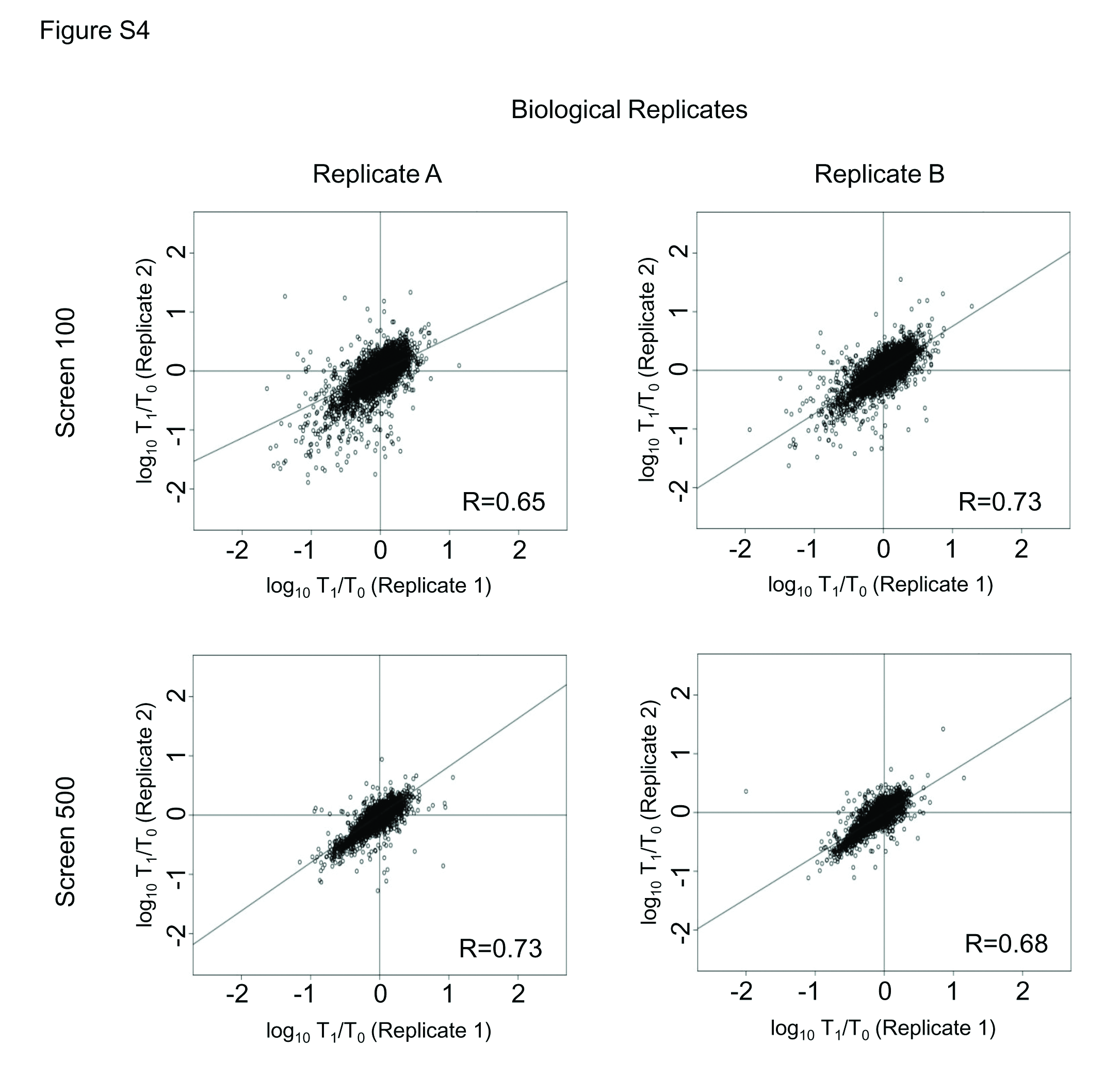

Supplement: Figure S4 — Scatter plots of log10(T1/T0) microarray data from the technical (PCR) replicates of the S100 and S500 viability screens. Amplification of the barcode sequence was performed in technical replicates (1 and 2) on gDNA isolated from each biological screen replicate (A and B). The amplification was limited to the exponential phase of PCR and the amount of input gDNA used corresponded to 100 template copies per shRNA for the S100 screen and 500 template copies per shRNA for the S500 screen. Pearson correlation values (R) for each graph are indicated in the boxes. Probes were filtered to remove those which did not pass a signal cutoff of greater than 2-fold median background in the T0 samples. (TIF) [file pone.0042341.s004.tif]

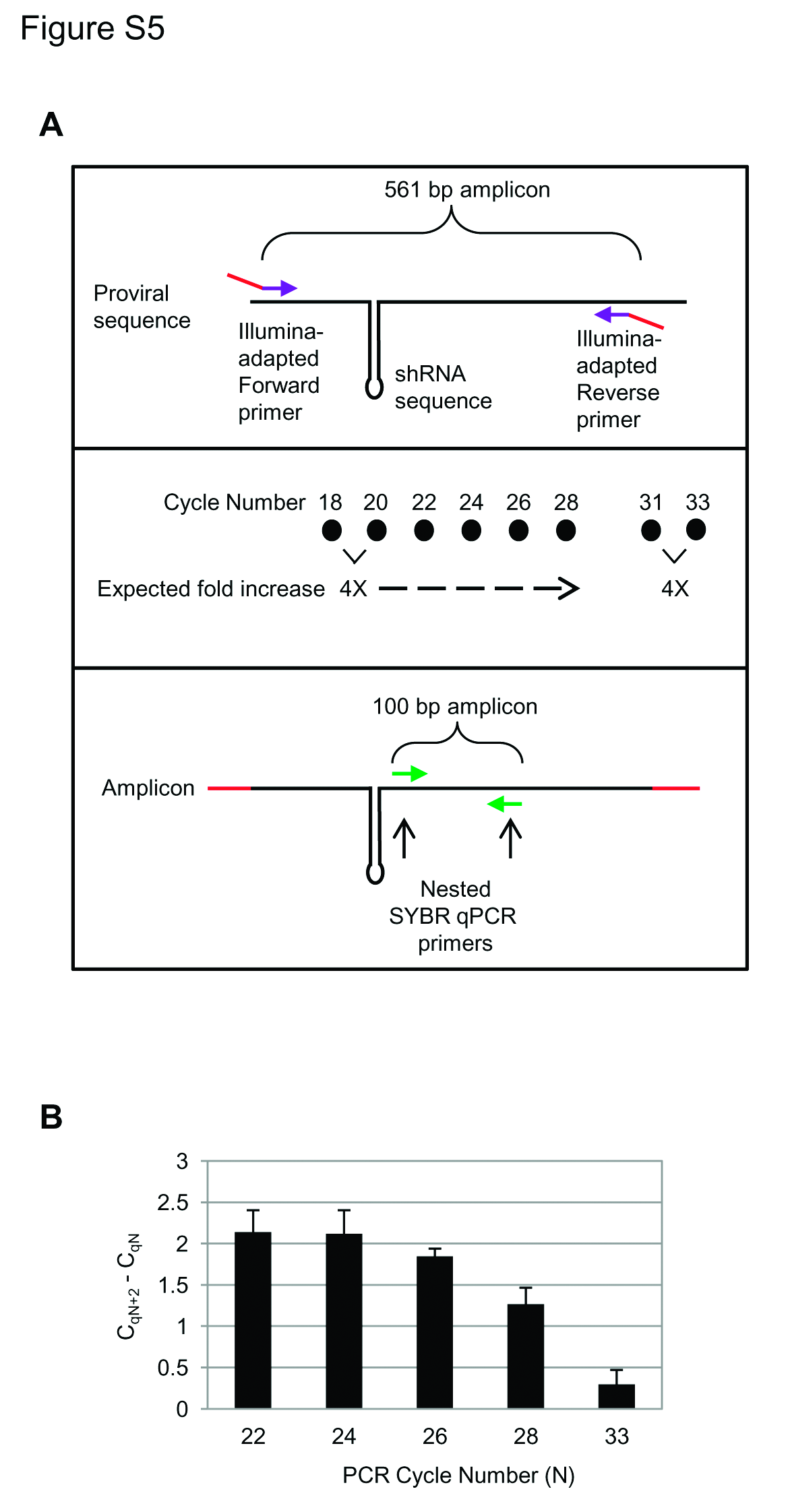

Supplement: Figure S5 — Identification of the exponential phase of PCR amplification of the shRNA sequences for NGS sample preparation. A. Schematic of the strategy used to identify the transition point from exponential to linear PCR amplification with the Illumina adapted primers that amplify the full-hairpin region. Replicate PCR reactions were prepared to amplify gDNA isolated from HEK293T cells transduced with the pooled shRNA library. A replicate reaction was stopped at the stated cycles. Subsequently, PCR products were used as templates for SYBR qPCR reactions using nested primers targeting a common sequence to examine the ΔCq between every other cycle. B. Graphical representation of the ΔCq for the qPCR reactions performed using diluted PCR samples (ΔCqN+2−ΔCqN) as a function of the Phusion PCR cycle number (N). (TIF) [file pone.0042341.s005.tif]
