# Supplementary material for: Optimized PCR Conditions and Increased shRNA Fold Representation Improve Reproducibility of Pooled shRNA Screens
Source: PLoS One. 2012 Aug 1;7(8):e42341. doi: 10.1371/journal.pone.0042341 (PMC3411659; doi:10.1371/journal.pone.0042341)
Supplement: Table S1 — PCR conditions affect reproducibility (DOCX) [file pone.0042341.s006.docx]

| **Table S1. PCR conditions affect reproducibility** | | | | | | | | |  |
| --- | --- | --- | --- | --- | --- | --- | --- | --- | --- |
| PCR cycles |  | 50 template copies per shRNA | | |  | 150 template copies per shRNA | | | |
|  |  | R (T_0_) | R (T_1_) | log_10_(T_1_/T_0_) |  | R (T_0_) | R (T_1_) | log_10_(T_1_/T_0_) | |
| 25 |  | 0.81 | 0.72 | 0.58 |  | 0.99 | 0.99 | 0.81 | |
| 30 |  | 0.86 | 0.81 | 0.49 |  | 0.99 | 0.98 | 0.69 | |
| Pearson correlation (R) values for reference sample (T_0_) and test sample (T_1_) and relative shRNA abundance measurement log_10_(T_1_/T_0_) for microarray data described in Figure 1. | | | | | | | | |  |
